# Serological Evidence of Common Equine Viral Infections in a Semi-Isolated, Unvaccinated Population of Hucul Horses

**DOI:** 10.3390/ani11082261

**Published:** 2021-07-30

**Authors:** Barbara Bażanów, Janusz T. Pawęska, Aleksandra Pogorzelska, Magdalena Florek, Agnieszka Frącka, Tomasz Gębarowski, Wojciech Chwirot, Dominika Stygar

**Affiliations:** 1 Division of Microbiology, Department of Pathology, Faculty of Veterinary Medicine, Wroclaw University of Environmental and Life Sciences, 50-375 Wroclaw, Poland; aleksandra.pogorzelska@upwr.edu.pl (A.P.); magdalena.florek@upwr.edu.pl (M.F.); 2 Centre for Emerging Zoonotic and Parasitic Diseases, National Institute for Communicable Diseases, Johannesburg 2131, South Africa; januszp@nicd.ac.za; 3 Grove Referrals, Fakenham NR21 8JG, UK; agnieszkafracka@gmail.com; 4 Department of Basic Medical Sciences, Faculty of Pharmacy with the Division of Laboratory Diagnostics, Wroclaw Medical University, 50-556 Wroclaw, Poland; tomasz.gebarowski@umed.wroc.pl; 5 Faculty of Veterinary Medicine, Wroclaw University of Environmental and Life Sciences, 50-375 Wroclaw, Poland; 106377@student.upwr.edu.pl; 6 Chair and Department of Physiology, School of Medicine with Dentistry Division in Zabrze, Medical University of Silesia, 40-055 Katowice, Poland; dstygar@sum.edu.pl

**Keywords:** Huculs, viral status, immunological status, equine viral diseases

## Abstract

**Simple Summary:**

The aim of the following research was an analysis of the occurrence of common equine viral infections in a Hucul herd, based on serological studies. This study provides epidemiological data concerning animals representing a semi-isolated herd, devoid of any specific prophylaxis. The obtained results provide a source of information on a primitive breed of horses, as well as giving an insight into viruses (among them, arboviruses) circulating within a local ecosystem.

**Abstract:**

Huculs (*Equus caballus*) are an old breed of primitive mountain horses, originating from the Carpathian Mountains. To the best of our knowledge, data concerning the epidemiology of viral infections observed within this breed are sparse. The objective of this study was to estimate the serological status of a semi-isolated, unvaccinated Hucul herd, with respect to both common equine viral infections and horse-infecting arboviruses, the presence of which was previously reported in Poland. Twenty horses of the Hucul breed, living in a remote area in Poland, were studied in 2018 from March to May. Using nasal secretion swabs as a specimen source, isolation attempts were negative regarding ERAV, EHV-1, EAV, and EIV. According to the virus neutralisation method, in the sera obtained from the animals, antibodies against the following viruses were detected: EHV-1 in 12 horses (60%; with titres from 1:8 to 1:64), EIV A/H7N7 in 13 (65%; titres from 1:20 to 1:80), EIV A /H3N8 in 12 (60%; titres from 1:20 to 1:80), USUV in 5 (25%; titres from 1:10 to 1:80), and ERAV in 1 (5%; titre 1:32). Antibodies against EAV, EIAV, and WNV were not present in the tested sera. The detected presence of specific antibodies associated with five out of the eight equine viruses investigated indicates that the Hucul herd, due to its partial separation and lack of specific prophylaxis, could serve as a sentinel animal group for the detection of equine viruses/arboviruses present within the local ecosystem. The detection of common equine viral infections within the herd provides additional epidemiological data concerning the breed.

## 1. Introduction

Huculs (*Equus caballus*), also known as Carpathian horses or Carpathian ponies, are a breed of primitive, small, mountain horses [1]. This old breed originates from the region of the eastern part of the Carpathian Mountains, and the first written material concerning these animals dates back to the beginning of the 17th century [1,2]. It is assumed that Huculs may be descendants of Tarpan horses mixed with Arabian horses, as well as Lipican, Hafling, Norik breed, and Fordwhich [2]. Since the Hucul horse is classified as a small-numbered breed, it has been a part of the FAO Program for the Preservation of Animal Genetic Resources [3]. Huculs are distinguished by their incredible strength, vitality, and resistance to harsh weather conditions; therefore, they can be kept on pastures throughout the year [1]. They are also known for their excellent health, including disease resistance, as well as for their high fertility and longevity [3,4]. The data concerning this breed are sparse (28 records in PubMed). To the best of our knowledge, epidemiological information in the form of only two papers exclusively reporting equine viral arteritis (EVA) infections is available for these animals [5,6].

The most common viral infections observed in equids are caused by viral agents belonging to different taxonomic families, including equine arteritis virus (EAV, *Arteriviridae*), equine herpesvirus 1 (EHV-1, *Herpesviridae*), equine rhinitis A virus (ERAV, *Picornaviridae*), and equine influenza A virus (EIV, *Orthomyxoviridae*) [7,8]. Additionally, when it comes to these animals, infections caused by Usutu virus (USUV, *Flaviviridae*), West Nile virus (WNV, *Flaviviridae*), and equine infectious anaemia virus (EIAV, *Retroviridae*) are of recently increasing importance [9,10].

Equine viral arteritis (EVA), caused by EAV, is a global infectious disease of horses characterised by abortion in pregnant mares. Other clinical signs like anorexia, depression, conjunctivitis, respiratory signs, and ocular discharge, as well as oedema of the eyelids, abdomen, prepuce and scrotum, or mammary glands, can also be observed [7,11]. Infection with EHV-1 is highly prevalent worldwide. Whereas it may be inapparent, in some animals it results in abortion or presents as acute rhinitis and pharyngitis, with the potential to spread into the more distal airways, leading to tracheobronchitis, bronchiolitis, and/or pneumonitis [7,12,13]. Other infections of the upper respiratory tract commonly observed in equids are caused by ERAV and the two main strains of EIV: equine-1 (H7N7) and equine-2 (H3N8) influenza A virus [14,15]. It is worth mentioning that the ERAV may also infect humans [16]. USUV and WNV are mosquito-borne zoonotic arboviruses transmitted by *Culex pipiens*, causing severe encephalitis and death in horses, birds, and humans [17]. The presence of antibodies to these two arboviruses, which were originally bound to Africa or Southeastern Asia, was previously confirmed in Poland in both humans and animals [9,18]. Bloodsucking insects, especially horseflies and deerflies, are EIAV vectors. In horses, symptoms of infections caused by the EIAV include high or recurrent fever, anaemia, weakness, and swelling of the lower abdomen, chest, legs, and scrotum, as well as weak pulse, irregular heartbeat, and abortion in pregnant mares. The latter disease presents no public health risk [19].

It was established that animal populations can serve as sentinels for the detection of environmental health threats [20]. According to the Centers for Disease Control and Prevention, the aim of sentinel surveillance is to obtain timely information in a relatively inexpensive manner, rather than to derive precise estimates of prevalence or incidence in the general population [20].

The objective of this study was to analyse the virological and immunological status of a herd of Hucul horses living in semi-natural conditions (and not vaccinated or artificially inseminated) with respect to common equine viral infections and horse-infecting arboviruses, the presence of which was previously reported in Poland. The method of maintenance and lack of medical intervention makes the studied herd, despite its small size, a useful model of sentinel animals/surveillance.

## 2. Materials and Methods

### 2.1. Ethics Statement

The blood samples and nasal swabs used in this project were taken for the routine diagnosis of EAV infections. The methods for the specimens’ collection were carried out in accordance with article 37ah–37ak of the Pharmaceutical Law act (Dz. U. z 2015 r. poz. 266, zmienione przez Dz. U. z 2019 r. poz. 499, 399 i 959 [Journal of Laws of the Republic of Poland from 2015, item 266 changed with Journal of Laws of the Republic of Poland from 2019, item. 499, 399 and 959]) from September 6th 2001 and the Experiments on Animals Act (Dz. U. 2019 poz. 1392 [Journal of Laws of the Republic of Poland 2019, item. 1392]) from 5 July 2019. In light of these regulations, approval of the Ethical Committee was not required. Informed consent was obtained from the owner of the animals.

### 2.2. Specimen

A herd of 20 Hucul horses in total (10 mares, 10 stallions) aged between 5 and 10 years, located in southwestern Poland (in Kalisz District, Greater Poland Voivodeship), was tested from March to May 2018. The animals had no contact with either other horses or wild equines, and they were kept on extensive pasture distant from inhabited areas throughout the year. None of the horses showed clinical symptoms at the time of sampling. There was no sign of clinical infection reported during the year prior to sampling, except for two incidents of abortion (for non-infectious reasons) and a single case of mild respiratory symptoms that was not diagnosed. All the horses were therefore considered healthy on the basis of physical examination and haematological and biochemical analyses (data not shown). None of the Huculs were vaccinated against any equine viral disease.

For serological tests, blood samples were collected from all the horses. Blood specimens were allowed to clot and were then centrifuged at 3000× *g* for 10 min, then the obtained sera were transferred into fresh tubes. Nasal swabs taken from the animals were transported to the laboratory in tubes containing EMEM supplemented with antibiotics, at 4 °C. The swabs were processed by pressing against the tube walls and vortexing, using the transport medium. The obtained fluid was centrifuged for 10 min at 3000× *g*. The secured supernatants were transferred into fresh tubes.

### 2.3. Virus Isolation

Attempted virus isolation of EAV, EHV-1, and ERAV from nasal swabs was performed using standard isolation procedures. Twenty-four-well polystyrene plates containing rabbit kidney (RK-13, ATCC^®^ CCL-37^TM^, ATCC, Manassas, VA, USA) or green monkey kidney (Vero, ATCC^®^ CCL-81^TM^) cell lines were inoculated with the processed swab specimens (50–100 μL into each well) [21]. Plates were incubated at 37 °C/5% CO_2_ and observed daily for 7–10 days, for the development of a cytopathic effect (CPE), using an inverted microscope (Olympus Corp., Hamburg Germany; Axio Observer, Carl Zeiss Microscopy Deutschland GmbH, Jena, Germany). In the absence of visible CPE, up to 5 blind subsequent passages were performed. Attempted virus isolation of EIV was carried out in embryonated chicken eggs (ECEs) [22]. The samples from nasal swabs were inoculated into the allantoic cavities of the ECEs. After inoculation, the ECEs were kept for 4–5 days in an incubator for chicken eggs at 38 °C and then cooled at 4 °C for 24 h, opened, and examined for the presence of any changes in the embryos and on the membranes. Allantoic fluid was harvested and tested by hemagglutination assay (HA) using red blood cells from chickens (RBC aggregation was regarded as a positive result).

### 2.4. Serology

Sera obtained from all the horses were tested for the presence of antibodies against EAV, EHV-1, ERAV, EIV (H7N7 and H3N8), USUV, WNV, and EIAV. Virus neutralisation (VN) tests were performed using the Bucyrus, Rac-H, and V1722/70 reference strains for the detection of EAV [23], EHV1 [24], and ERAV [25], respectively. In order to detect antibodies to EIV, a haemagglutination inhibition test (HI) was carried out according to the World Health Organisation (WHO) recommendations [26] using A/equine/Miami/1/63 (H3N8) and A/equine/Prague/1/56 (H7N7) reference strains.

A microneutralisation assay was applied for the detection of antibodies against USUV and WNV, using the previously described procedure [9,27]. The presence of precipitating antibodies against the EIAV gag p26 protein [28,29] was tested via the agar gel immunodiffusion test (AGID), employing a commercial Equine Infectious Anemia Antibody Test Kit LAB-EZ /EIA (Zoetis, Parsippany, NJ, USA).

All the VN and HI tests were carried out in the presence of control sera (positive and negative) from the in-house collection.

### 2.5. Statistical Analysis

Statistical analysis was conducted in PQStat version 1.6.8 (PQ Stat Software, Poznań, Poland) at a significance level of 5% using Fisher’s test. The correlation between the age and seropositivity to individual pathogens was evaluated. For this purpose, the animals were divided by age into two groups: 5 years old (group of youngest horses in the herd) and over 5 years old.

## 3. Results

According to the results of the virus isolation, all the nasal swabs were negative for the presence of EAV, EHV-1, ERAV, and EIV. Specific antibodies were detected for five out of the eight examined equine viruses. Detailed results of our serological testing are shown in Table 1 and Table 2.

Of the 20 Huculs tested, 12 (60%) were positive for antibodies to EHV-1 with titres ranging from 1:8 to 1:32. Antibodies to equine influenza viruses A/H7N7 and A /H3N8 were found in the sera of 13 (65%) and 12 (60%) horses, respectively, with titres ranging from 1:20 to 1:80 against both viruses. USUV seropositivity was detected in five horses (25%) with titres ranging from 1:10 to 1:80. Only one (5%) animal had antibodies to ERAV (titre of 1:8). None of the horses had detectable antibody levels for EAV, EIAV, or WNV.

Statistical analysis showed no significant correlations between the variables of age group and antibody presence for viruses EHV1 (*p* > 0.999), ERAV (*p* > 0.999), H7N7 (*p* = 0.3498), H3N8 (*p* = 0.1698), and USUV (*p* > 0.999). Due to the negative results of the serological investigation, statistical analyses for age vs. antibody presence for EAV, WNV, and EIAV were not performed.

## 4. Discussion

Nasal swabs obtained from all the horses tested in this study were negative for the presence of the four common equine viruses investigated, which indicates the absence of ongoing infection with EAV, EHV-1, ERAV, or EIV at the time of swabbing.

Serum neutralising antibodies following infection with EAV have been shown to persist for years, and it was postulated that humoral immunity to this virus is long-lasting, if not lifelong [5,7]. Thus, the absence of detectable anti-EAV antibodies in the horses tested in our study indicates that they had no previous exposure to the virus, for which the main route of infection is direct contact (although spread through fomites has also been documented) [6]. Compared with our results, analysis of the largest Hucul stud in Poland (where the animals are commercially used), performed by Rola et al. in the years 2006–2008, showed the presence of antibodies to EAV in 55.1% of the horses [5]. Subsequent analysis concerning the same stud, carried out between the years 2010 and 2013, confirmed the presence of anti-EAV antibodies in 38% of the collected samples [6]. With respect to the total equid population in Poland, antibodies against EAV were detected in around 20% of the tested horses [30], and EVA was identified as a cause of 23% of abortions in mares [31]. The EAV seroprevalence reported in Europe is highly diverse. The lowest ratio was observed in the U.K. (1.3%). Reference countries in western and central Europe presented a range of seroprevalence from 14% to 20%, while in eastern Europe, it oscillated within the range of 14–75% [32,33,34,35,36,37].

EHV-1 is highly prevalent, spreads quickly among horses, and establishes a latent carrier state, which may possibly last for life. In latently infected animals, stressors, e.g., transport or pregnancy, can induce respiratory viral shedding [12,13]. The percentage of horses seropositive for EHV-1 in the population of southeastern Poland’s breeding farms, stallion herds, purchasing centres, and riding clubs ranges from 75% to 100% [38]. Compared to the situation in Poland, in Spain, only 53.9% of horses tested seropositive for EHV-1, while in the Netherlands, this proportion was even smaller, as 28.2% of the animals were positive [39,40]. In the present study, antibodies against EHV-1 were detected in 60% of the Huculs tested. Taking into consideration the facts that the spread of the virus requires direct contact and clinical symptoms suggesting viral infection were not observed within the herd, we assume that the virus might have been introduced by a previously latently infected animal.

Antibodies to ERAV were detected in only 5% of the horses tested. This relatively low level of ERAV infections is probably the result of the separation of the tested herd. In Poland, high ERAV seropositivity (72%) was reported in different breeds of horses [8]. In England, Switzerland, and Denmark, the observed ERAV seropositivity levels ranged from 2.3% to 14%; contrastingly, research showed that in Germany and the Netherlands, these values were far higher (39% to 90%) [14,16,41,42].

In the present study, a high percentage of EIV seropositivity was detected against subtypes H7N7 (65%) and H3N8 (60%). Since the first isolation in 1963 in the USA, the subtype H3N8 (Miami 63) of EIV has continued to circulate among horses, causing epidemics and minor outbreaks of equine influenza respiratory disease in Europe and North America [15,43,44]. The last major outbreak caused by H3N8 in Europe took place in 2018/2019 and involved the U.K., Ireland, France, Germany, and Belgium. While in Europe, the most commonly found is Florida clade 2, the recently reported EIV infections were caused by Florida clade 1, which had, until then, been circulating mainly in the United States [45,46].

The EIV H7N7 subtype (Prague 56) was first isolated in 1956 from horses in Eastern Europe [47]. This subtype has not been isolated since the last outbreak recorded in 1979 and was thought to be extinct. However, antibodies to this subtype are still detectable in horses, suggesting that it still circulates in equine populations [44]. During the last decade of the 20th century, H7N7-positive horses were reported in Belgium, Russia, the USA, India, and Nigeria [44,48,49]. Interestingly, in their study, Guo et al. reported that there was cross-reactivity observed within the H7 subtype influenza virus. The cross-reactivity was detected between H7N9 and subtypic viruses H7N2, H7N3, and H7N7 [50]. An analysis of khulan (*Equus hemionus hemionus*) in Mongolia, performed in 2015, showed that two of the tested animals were positive for the H7 virus type. While cross-reaction with the H7N9 strain was excluded in both cases, antibodies obtained from the horses were assigned as H7N7 using a protein microarray (PA) technique, but when one of the sera was additionally tested via single radial haemolysis assay, it proved to be H7N3 positive. The authors suggested co-circulation of both subtypes, which are of equine and avian origin, respectively [51]. Antibodies to the H7 protein were also detected in the same animal species in Nigeria [52], where a haemagglutination inhibition technique using H7N3 antigen was applied. Taking into the consideration the results of the above-mentioned investigations, the positive sera obtained in our study may represent remains of the circulating H7N7 equine-originating virus or the presence of other H7 subtypes, most probably of avian origin. The number of animals seropositive to both EI viruses among the Huculs tested in the present study was surprisingly high, particularly regarding the fact that these horses were not vaccinated against equine influenza and were living in isolation, without contact with other domestic animals. In the late 1980s in China, an outbreak caused by the H3N8 virus strain more closely related to avian H3 than to the equine H3 influenza virus was described [48]. Moreover, analysis of the outbreak of EI in New South Wales in 2007 suggested the potential role of birds as mechanical spreaders of the equine H3N8 virus [53]. Both scenarios equally explain the results of our investigation, yet other mechanisms of transmission of the EI viruses to the herd tested in the present study cannot be excluded.

In Europe, the USUV was isolated for the first time from bats in Germany, and the first human infection with this virus took place in Italy in 2009; three years later, in Germany, research showed that 1 out of 4200 patients had antibodies against USUV [18,54,55]. The relatively high USUV seropositivity detected in the present study corroborates the results of a survey recently carried out in Poland, where 27.98% of the tested horses were seropositive to this virus [9]. However, these results are quite different from the findings of a study conducted in Croatia, where USUV-specific neutralising antibodies were detected in 2 of the total 1380 animals [56]. The seroprevalence of USUV in European horses is highly varied. In Serbia, it was found to be only 0.3%, and in Spain, it was 1.4%; however, in Italy it was as high as 89.2% [57]

The first report of the appearance of WNV in Poland comes from the years 1995–1996, when Juricova et al. conducted research on two sparrow species and stated a relatively high prevalence (2.8% in house sparrow (*Passer domesticus*) and 12.1% in Eurasian tree sparrow (*Passer montanus*)) of anti-WNV antibodies in the sera of the tested birds [58]. In 2013 in the Czech Republic, Rudolf et al., while testing mosquitoes, found WNV strains that were genetically similar to those isolated in Austria, Italy, and Serbia, which may testify to the spreading of this virus across all of Europe [18]. In studies performed in Poland, antibodies to WNV were reported in 35.7% of avian sera [9]. Two serological surveys concerning the prevalence of WNV in horses in Poland showed the presence of the antibodies in 0.65% of sera collected before 2008 and in 15.08% of samples obtained in the years 2012–2013 [9,30]. On the contrary, all the Huculs examined in the present study were negative for anti-WNV antibody presence. WNV infections have also been confirmed in various European countries like Serbia, Croatia, Greece, Slovakia, Spain, and Albania, and the seroprevalence of this virus in horses was found to range from 2.75% to 22% [59].

The absence of detectable levels of antibodies to EIAV observed in the present study was an expected result, considering the fact that since the last confirmed case of EIAV in Poland was in 1960, and only two additional cases were reported in 2015 [60]. Between 2007 and 2014, however, outbreaks of the disease were reported in Belgium, Bosnia, Croatia, France, Germany, Greece, Hungary, Ireland, Italy, Latvia, Romania, Serbia, Slovenia, and the United Kingdom [10], indicating a need for ongoing monitoring of the disease’s spread.

The serological data obtained for the herein-analysed small group of Hucul horses, especially information concerning the absence of antibodies against certain viruses (EAV, EIAV, and WNV), cannot be extrapolated with regard to the whole Hucul population in Poland (over 1500 animals in total [2]). In order to understand the epidemiological status of the breed in our country in a statistically appropriate manner, a larger number of analyses need to be performed.

## 5. Conclusions

The present study provides useful epidemiological data regarding viral infections in Hucul horses, supplementing the sparse information on the matter available to date. Partial isolation of the tested herd, in addition to the fact that it was also devoid of any specific prophylaxis, allowed us to analyse the circulation within a local ecosystem of viruses causing infections in horses. Among these investigated viruses were those transmitted via vectors. The fact that the tested animals were apparently healthy at the moment of sampling and throughout the year prior suggests that the detected antibodies are a result of earlier exposures, but it may also be related to the natural resistance of Huculs, as indicated by other authors [1,5]. In such a case, in order to recognise the epidemiology of viral infections concerning the breed, further serological surveys are required.

## Figures and Tables

**Table 1:** Results of serological investigation of the Hucul herd.

Virus Tested	Number of Positive Results/Number of the Horses Tested	Percentage of Seropositive Horses (%)
equine arteritis virus	0/20	0
equine herpes virus 1	12/20	60
equine rhinitis A virus	1/20	5
equine influenza A virus H7N7	13/20	65
equine influenza A virus H3N8	12/20	60
Usutu virus	5/20	25
West Nile virus	0/20	0
equine infectious anaemia virus	0/20	0

**Table 2:** The immune status in individual horses.

No.	Sex	Age (Years)	Obtained Titres of Antibodies							
			EAV	EHV1	ERAV	H7N7	H3N8	USUV	WNV	EIAV
1	mare	10	0	0	0	40	20	40	0	0
2	mare	9	0	16	0	0	20	80	0	0
3	mare	5	0	0	0	20	0	20	0	0
4	mare	10	0	32	8	0	0	20	0	0
5	mare	5	0	0	0	20	0	0	0	0
6	mare	8	0	0	0	0	80	0	0	0
7	mare	10	0	0	0	20	20	0	0	0
8	mare	5	0	8	0	40	40	0	0	0
9	mare	5	0	32	0	40	40	0	0	0
10	mare	9	0	32	0	0	40	0	0	0
11	stallion	5	0	0	0	80	40	0	0	0
12	stallion	6	0	32	0	20	0	10	0	0
13	stallion	6	0	8	0	20	20	0	0	0
14	stallion	5	0	32	0	0	0	0	0	0
15	stallion	5	0	16	0	20	0	0	0	0
16	stallion	5	0	0	0	0	0	0	0	0
17	stallion	9	0	16	0	20	20	0	0	0
18	stallion	10	0	8	0	0	20	0	0	0
19	stallion	9	0	16	0	20	0	0	0	0
20	stallion	5	0	0	0	20	20	0	0	0

## Data Availability

The original data are available by contact with the corresponding author.
